# Does non-motorists’ safety perception of autonomous vehicles vary across opinion change stemming from crash occurrence? Investigating perceptions using fixed and random parameter ordered logit models

**DOI:** 10.1016/j.heliyon.2023.e19913

**Published:** 2023-09-06

**Authors:** Abimbola Ogungbire, Srinivas S. Pulugurtha

**Affiliations:** a Infrastructure & Environmental Systems (INES) Program, The University of North Carolina at Charlotte, 9201 University City Boulevard, Charlotte, NC 28223-0001, USA; b Civil & Environmental Engineering Department, The University of North Carolina at Charlotte, 9201 University City Boulevard, Charlotte, NC 28223-0001, USA

**Keywords:** Autonomous vehicle, Crash, Safety, Perception, Pedestrian, Bicyclist

## Abstract

The perception of non-motorists toward autonomous vehicles (AVs) could change or remain the same after hearing about a fatal AV crash. This study aims to discern the differences between non-motorists whose opinions were influenced and those whose views remained unchanged after hearing about the fatal AV crash. Additionally, this study investigates how the operational competence of AV manufacturers affect non-motorist's safety perception. A fixed parameter ordered logit model was explored to explain non-motorists’ safety perception for respondents who changed their opinion after a crash occurrence and those who did not. A random parameter ordered logit model was then explored to address the difference in residual variance. The finding from the random parameter ordered logit model indicates that two factors (i.e., future impact of AVs on traffic injuries and willingness to adopt Pittsburgh's streets as a proving ground) were significant for the respondents with negative change in opinion after the crash. The result also suggests that negative information such as the crash in Arizona may influence non-motorists’ safety perception of AVs, but this effect can be mitigated by providing positive information about the AVs. The results also demonstrate that non-motorists who perceive the operational competence of the AV manufacturers to be high have higher safety perception of the AVs, while those who perceive the operational competence of the AV manufacturers to be low have lower safety perception of the AVs. This study offers recommendations for policies and interventions to enhance non-motorists' safety based on the examination of factors associated with safety perception among respondents who changed their opinion following a crash and those who maintained their initial stance.

## 1 Introduction & background

The plan to introduce autonomous vehicles (AVs) into the transportation system is gaining increasing attention worldwide. Its potential to transform the way people travel makes it even more anticipated. While some people are excited to see what this technology can do, others are worried about its potential downsides and the danger it might bring [[1], [2], [3]]. There is an expected sway in the opinions of the public (road users and potential buyers of AVs) with the penetration of AVs and event occurrence (e.g., crash involving an AV) [4,5]. Therefore, it is important to understand the safety perception of non-motorists (cyclists and pedestrians) that will eventually share roads with AVs.

Many industry experts are confident in improving road safety with the transition to AVs [[4], [5], [6]]. The findings from past research showed that 94% of automobile crashes are due to human errors [7]; thus, eliminating human error will reduce crash fatalities by as much as 94% [8]. When non-motorists perceive an increase in the danger associated with AVs due to crashes, their overall safety perception of AVs tends to decrease. Consequently, this decline in safety perception can lead to hesitation or resistance in passing laws and policies that support the widespread adoption of AVs. As a result, the penetration of AVs into society may be hindered.

Past studies found that people in the western world do not trust the AV technology [9,10], nor are they comfortable with the safety of non-motorists in an AV-driving environment [11]. In 2018, a self-driving car killed Elaine Herzberg, who was wheeling her bicycle in Tempe, Arizona. The fatal crash of the pedestrian generated media attention, creating a vast reaction from the public, practitioners, and industrialists [12]. In other words, death raises many ethical and proto-legal questions in the progressive autonomous field [11]. This crash has influenced several non-motorists’ opinion about sharing the road with AV. Therefore, further research is necessary to gain insights into how non-motorists’ safety perception of AVs would be affected in the event of a crash occurrence.

Several studies have been carried out on the general perception of the public about AVs. Many of these studies focused on the acceptance and receptivity of AVs while a few studies have explored the safety perception of the public about AVs [[12], [13], [14], [15], [16]]. According to the safety perception and acceptance of AVs from a study conducted in 2018, AVs were perceived as a less risky form of transportation than human-driven vehicles [16]. However, the perception could vary for passengers and non-motorists. There is no doubt that non-motorist's willingness to share the road with AV will play a crucial role in the widespread implementation of this technology. The result from a survey conducted in 2017 shows that pedestrians or bicyclists with direct interaction with AVs have a higher expectancy of safety [12]. However, a comprehensive review of the literature shows mixed trends and differences in AV perception and acceptance.

While the current discourse on AV related studies identify 'trust in AV among the public users' as a key determinant in assessing the acceptance of AV, there are only a few studies that give a breakdown of how the ‘trust’ construct is being distributed. Choi & Ji [15] proposed a three-dimensional trust construct in AV: (i) system predictability (the belief that the system is predictable); (ii) technical competence (the belief that the system runs effectively and accurately); and, (iii) situation management (the belief that human can override the system if needed) [15]. However, 'the perceived operational competence of the AV manufacturers, i.e., the belief that AV manufacturers can effectively manage AVs during a trip, depends on their safety, performance, and other important information. This would involve having the necessary knowledge and resources to manage AVs efficiently, as well as the ability to develop strategies and plans that consider all the necessary information. Ultimately, operational competence is key to ensuring that AVs are managed well, and that safety and performance are taken into consideration. This study investigates how respondents' perceived operational competence of AV manufacturers could affect their safety perception of AVs.

The pedestrian fatality case involving a self-driving vehicle may have changed the perception of some AV enthusiasts and left them wondering if this technology would do more harm than good. For others, their opinions about AVs could have remained the same. Past studies did not provide insights into the possibility of identifying people whose opinions could be impacted by safety concerns in AVs. It is hard to tell how the attitude and beliefs of non-motorists could influence their safety perception of AVs after hearing about a crash involving an AV and a non-motorist. It is important to research and understand how these attitudes and beliefs differ for these two types of non-motorists. This study investigates how non-motorists whose opinion swayed differ from non-motorists whose opinions remain unaffected by hearing about the crash. The premise is that these two kinds of people have different safety perceptions of AVs (i.e., they have different beliefs and attitudes toward using them). This study highlights the determinants of safety perception among these two distinct groups of non-motorists: those whose opinions were altered hearing about the crash and those whose views remained unswayed.

Overall, the study aims to: a) study the opinion of non-motorists regarding their trust in the AV technology; and study the association between trust in the AV technology and their perceived willingness to share the road with an AV; and, b) identify the factors responsible for non-motorist's safety perception among users whose opinions were influenced and those who hold the same opinion after hearing about the crash.

### 1.1 Effect of perceived operational competence of AV manufacturers on public's safety perception

While the development and deployment of AVs have been a topic of extensive research, studies specifically examining the perceived operational competence of AV technology and its impact on public willingness to share the roads with these vehicles are notably limited. While there is ample research on the general perception of AVs, relatively few studies have focused on the perceived operational competence of AV technology. For instance, one of the few studies in this domain was conducted by Hurst & Sintov [17], who found that perceived competence of AV manufacturers significantly influenced consumer acceptance of AVs. Approximately one third of individuals harbor misconceptions regarding the availability and operational capability of AVs [18]. Liu et al. [19] postulated that those who erroneously believe that a mature business infrastructure has been established around AVs typically exhibit a more favorable attitude towards their use. Understanding how the perceived operational competence of AV manufacturers influences willingness to share the road with AVs requires insights into the underlying mechanisms. Though some studies, such as that of Choi & Ji [15], have suggested that the trust in AV manufacturers mediates the relationship between perceived competence and willingness to ride, this area remains largely unexplored. This perception of competence encapsulates the perceived ability of AV manufacturers to develop, deploy, and manage AVs effectively and safely. Given the importance of safety and efficiency in the public's acceptance of AVs, it is plausible to hypothesize that the higher the perceived operational competence of AV manufacturers, the greater the public's willingness to use these vehicles.

Understanding how the perceived operational competence of AV manufacturers influences safety perception and willingness to use these vehicles will provide invaluable insights for the industry, helping to shape development strategies and public outreach efforts. Moreover, exploring the underlying mechanisms will provide a better understanding of the factors that influence AV acceptance.

### 1.2 Effects of positive & negative news on safety perception of AV

The effect of positive and negative news on non-motorists' safety perception of AVs is a topic of significant importance as AV technology continues to develop and shape the future of transportation. Positive news about AVs can have several beneficial effects on non-motorists' safety perception. Past studies have shown that exposure to positive news coverage, highlighting successful deployments, improved safety features, and benefits to non-motorists, can increase familiarity and trust in the AV technology [20,21]. Positive news that emphasize a reduction in the number of crashes, enhanced traffic flow, and improved safety measures can contribute to a positive perception of AV safety among non-motorists [22]. Additionally, positive news coverage addressing non-motorists' concerns, such as ethical considerations, cybersecurity, and accessibility, can alleviate anxieties and improve the safety perception [23,24]. The overall impact of positive news tends to foster familiarity, trust, and enhanced safety perception of AVs among non-motorists.

On the other hand, negative news about AVs can significantly impact non-motorists' safety perception [25,26]. Negative news that focusses on crashes, technological failures, and ethical dilemmas associated with AVs can heighten non-motorists' safety concerns and increase perceived risks [12]. Fear and mistrust can arise from negative news coverage that portrays AVs as unpredictable or unreliable, negatively affecting safety perception [18]. It is important to note that the impact of negative news may be more pronounced than that of positive news, as negative information tends to attract more attention and generate stronger emotional responses.

It is likely that the fears generated by negative news often outweigh the positive effects. However, achieving a balance between positive and negative news coverage is crucial in shaping non-motorists' overall safety perception of the AVs. To further explore the influence of negative news on non-motorists' safety perception of the AVs, this study aims to identify the factors associated with safety perception among users whose opinions were influenced, as well as those who maintained the same opinion after the occurrence of a crash involving an AV.

## 2 Data & method

### 2.1 Data description

In response to the testing of AVs in Pittsburgh since 2016 and a desire to understand public sentiment towards these vehicles, a survey was designed and conducted in 2017 and subsequently in 2019 by BikePGH (a charitable non-profit organization working towards making the Pittsburg community safer, accessible and bicycle friendly) [14]. The 2017 survey, which aimed to capture feelings towards sharing the road with AVs as bicyclists and pedestrians, was initially launched to donor-members, yielding 321 responses. In 2019, the survey was repeated with 796 participants, with an added focus on regulations and demographics. Both surveys intended to gather public perceptions and experiences interacting with AVs, contributing to an understanding of public sentiment towards this emerging technology. The follow-up survey in 2019 aimed to explore changes in public sentiment over time and ensure the safe integration of AVs in shared spaces [14]. For this study, the 2019 survey was used. The questions asked are listed in Table 1.

**Table 1:** Survey questions collected by BikePGH in 2019.

Indicator ID	Indicator	Question	Response
DV	Safe with AV	On a typical day, how safe do you feel sharing the road with AVs?	1 being very unsafe and 5 being very safe
Q1	Familiarity with AV through news	What effect do you think that AVs will have on traffic injuries and fatalities?	Not at all; to a little extent; to some extent; to a moderate extent; to a large extent
Q2	Familiarity with AV technology	How familiar are you with the technology behind AVs?	Not familiar; somewhat familiar; mostly familiar; extremely familiar
Q3	Shared cyclist	Have you shared the road with an AV while riding your bicycle on the streets of Pittsburgh?	Not sure; no; yes
Q4	Safe with human driven vehicle	On a typical day, how safe do you feel sharing the road with human-driven cars?	1 being very unsafe and 5 being very safe
Q5	Speed threshold of AV at 25 mph	On City of Pittsburgh public streets, should AV speeds be capped at 25 mph when operating in autonomous mode?	Not sure; no; yes
Q6	Have a smartphone	Do you own a smartphone?	No; yes
Q7	Manual mode of AV in school zones	On the city of Pittsburgh public streets, do you think that AVs should operate in manual mode while in an active school zone?	Not sure; no; yes
Q8	Share trip data with authorities	On the city of Pittsburgh public streets, should AV manufacturers be required to share some non-personal data (e.g., number of trips; pick up/drop off locations; number of miles driven) with the proper authorities (e.g., Department of Mobility; PennDOT; Public Safety)?	Not sure; no; yes
Q9	BikePGH member	Are you currently an active member of BikePGH?	No; yes
Q10	Pittsburgh public street as a proving ground	What do you think about the use of Pittsburgh's public streets as a proving ground for AVs?	Disapprove; somewhat disapprove; neutral; somewhat approve; approve
Q11	Impact of AV	What effect do you think that AVs will have on traffic injuries and fatalities?	Significantly worse; slightly worse; no effect; slightly better; significantly better
Q12	Shared pedestrian	Have you been near an AV while walking or using a mobility device (wheelchair, etc.) in Pittsburgh?	Not sure; no; yes
Q13	Opinion on Arizona crash	In March of 2018, an AV struck and killed Elaine Herzberg, a pedestrian, in Tempe, AZ. As a pedestrian and/or bicyclist, how did this event and its outcome change your opinion about sharing the road with AVs?	Negative opinion; no change; positive opinion
Q14	Two employees for AV	On the city of Pittsburgh public streets, should AVs always have two full-time employees (pilot and co-pilot)?	Not sure; no; yes
Q15	Auto ownership	Do you (or someone in your household) own an automobile?	No; yes
Q16	Share AV performance data	On the city of Pittsburgh public streets, should AV manufacturers be required to disclose information and data as to the limitations; capabilities; and real-world performance of their cars with the proper authorities?	Not sure; no; yes
Q17	Report safety concerns to the authorities	On the city of Pittsburgh public streets, should AV manufacturers be required to report all safety-related incidents with the proper authorities; even if a police report isn't required?	Not sure; no; yes
Q18	Age	What is your age?	Under 18; 25–34; 35–44; 45–54; 55–64; 65+

The dataset was summarized across the change in the opinion of respondents about AVs after an automated car operating in self-driving mode killed a pedestrian in Arizona. The substantial differences in respondents' demography and experiences/expectations of AVs among the three groups of opinions after the crash in Arizona are summarized in Table 2.

**Table 2:** Descriptive statistics of data.

Question/Response	Levels	Frequency			
		Negative Opinion (n = 263)	No Change (n = 442)	Positive Opinion (n = 21)	$\chi^{2}$
Familiarity with AV through news	To a large extent	80 (30.4%)	173 (39.1%)	12 (57.1%)	26.61***
	To a moderate extent	92 (35.0%)	167 (39.1%)	7 (33.3%)	
	To some extent	70 (26.6%)	78 (17.6%)	1(4.8%)	
	To little extent	21 (8.0%)	16 (3.6%)	1(4.8%)	
	Not at all	0(0.0%)	8 (100.0%)	0(0.0%)	
Familiarity with AV technology	Extremely familiar	40 (15.2%)	103 (23.3%)	8(38.1%)	30.43***
	Mostly familiar	77 (29.3%)	147 (33.3%)	12(57.1%)	
	Somewhat familiar	112 (42.6%)	163 (39.9%)	1 (4.8%)	
	Not familiar at all	34 (12.9%)	29 (6.6%)	0 (0.0%)	
Shared cyclist	Yes	142 (54.0%)	245 (55.4%)	12 (57.1%)	2.67
	No	85(32.3%)	136(30.8%)	4 (19.0%)	
	Not sure	36 (13.7%)	61 (13.8%)	5 (57.1%)	
Safe with human driven vehicle	5 = very safe	5 (1.9%)	12 (2.7%)	0 (0.0%)	2.78
	4	39 (14.8%)	65(14.7%)	1 (4.8%)	
	3	118 (44.9%)	198 (44.8%)	11 (54.4%)	
	2	78 (29.7%)	129 (29.2%)	7 (33.3%)	
	1 = very unsafe	23 (8.7%)	38 (8.6%)	2 (9.5%)	
Speed threshold of AV at 25 mph	Yes	155 (58.9%)	119 (26.9%)	1 (4.8%)	102.93***
	No	39 (14.8%)	200 (45.2%)	15 (71.4%)	
	Not sure	69 (26.2%)	123 (27.8%)	5 (23.8%)	
Have a smartphone	Yes	249 (94.7%)	431 (97.5%)	21 (100%)	4.76
	No	14 (5.3%)	11 (2.5%)	0 (0.0%)	
Manual mode of AV in school zone	Yes	177 (67.3%)	175 (39.6%)	47 (19.0%)	66.7***
	No	34 (12.9%)	147 (33.3%)	12 (57.1%)	
	Not sure	52(19.8%)	120 (27.1%)	5 (23.8%)	
Share trip data with authorities	Yes	222 (84.4%)	309 (69.9%)	13 (61.9%)	23.84***
	No	18(6.8%)	67 (15.2%)	6 (28.6%)	
	Not sure	23(8.7%)	66(14.9%)	2(9.5%)	
BikePGH member	Yes	126 (47.9%)	227 (51.4%)	12 (57.1%)	1.19
	No	137 (52.1%)	215 (48.6%)	9 (42.9%)	
Pittsburgh public street as a proving ground	Approve	52 (19.8%)	287 (64.9%)	19 (90.5%)	192.5***
	Somewhat approve	72 (22.4%)	84(19.0%)	1 (4.8%)	
	Neutral	35 (13.3%)	44 (10%)	1 (4.8%)	
	Somewhat disapprove	60 (22.8%)	17 (3.8%)	0 (0.0%)	
	Disapprove	44 (16.7%)	10 (2.3%)	0 (0.0%)	
Impact of AV	Significantly better	47 (17.9%)	220 (49.8%)	16 (76.2%)	128.18***
	Slightly better	94 (35.7%)	152(34.4%)	4 (19.0%)	
	No effect	48 (18.3%)	46 (10.47%)	1 (4.8%)	
	Slightly worse	46 (17.5%)	18 (4.1%)	0 (0.0%)	
	Significantly worse	28 (10.6%)	6 (1.4%)	0 (0.0%)	
Shared pedestrian	Yes	155 (58.9%)	286(64.7%)	10 (47.6%)	4.83
	No	86 (32.7%)	126(28.5%)	8 (38.1%)	
	Not sure	22 (8.4%)	30(6.8%)	3 (14.3%)	
Safe with AV	5 = very safe	21 (8.0%)	185 (41.9%)	14 (66.7%)	163.67***
	4	73 (27.8)	154 (34.8%)	5 (23.8%)	
	3	90 (34.2%)	75 (17.0%)	1 (4.8%)	
	2	44 (16.7%)	14 (3.2%)	1 (4.8%)	
	1 = very unsafe	35 (13.3%)	14 (3.2%)	0(0.0%)	
Two employees for AV	Yes	136(51.7%)	110(24.9%)	4(19.0%)	64.47***
	No	68 (25.9%)	213 (48.2%)	15 (71.4%)	
	Not sure	59 (22.4%)	119 (26.9%)	2(9.5%)	
Own a vehicle	Yes	247 (93.9%)	419 (94.8%)	21 (100%)	1.48
	No	16 (6.1%)	23 (5.3%)	0 (0.0%)	
Share AV performance data	Yes	248 (94.3%)	355 (80.3%)	16 (76.2%)	30.21***
	No	10 (3.8%)	44 (10.0%)	4 (19.0%)	
	Not sure	5 (1.9%)	43 (9.7%)	1 (4.8%)	
Report safety concerns to the authorities	Yes	252 (95.8%)	348 (78.7%)	15 (71.4%)	41.23***
	No	4 (1.5%)	54 (12.2%)	4 (19.0%)	
	Not sure	7 (2.7%)	40 (9.0%)	2 (9.5%)	
Age	Under 18	1 (0.4%)	0 (0.0%)	0 (0.0%)	10.18
	18–24	7 (2.7%)	18 (4.1%)	2 (9.5%)	
	25–34	72 (27.4%)	112 (25.3%)	4 (19.0%)	
	35–44	65 (24.7%)	95 (21.5%)	6 (28.6%)	
	45–54	43 (16.3%)	76 (17.2%)	2 (9.5%)	
	55–64	49 (18.6%)	78 (17.6%)	5 (23.8%)	
	65+	26 (9.9%)	63 (14.3%)	2 (9.5%)	

N.B: *indicate significant $\chi^{2}$- statistic with p-value <0.05; **indicate significant $\chi^{2}$- statistic with p-value <0.01: ***indicate significant $\chi^{2}$- statistic with p-value <0.001; and $\chi^{2}$ is the Chi-Square statistic comparison among the negative change, no change and positive change in opinion.

The data indicates that a significantly higher proportion (57.1%) of the respondents who pay attention to AVs in the news to a large extent have a positive opinion about AVs. The respondents who pay no attention to AVs in the news did not change their opinion, whereas those who pay attention to some extent have a negative opinion. A greater proportion of the respondents familiar with the technology (38.1%–57.1%) have a positive opinion, while those unfamiliar (12.9%) had a negative opinion. Owning a technology (smartphone and private car) indicates a higher proportion (100%) of positive opinion about AVs, while the opinion is insignificantly higher among the respondents without these technologies. A greater proportion of the respondents who feel safe sharing the road with the AVs have a positive opinion about the AVs, compared to those who feel unsafe or indifferent.

A greater proportion of the respondents who want manual operation in school zones have a negative opinion, while those who are unsure or do not want manual operation of AVs in school zones have no significant change in the opinion. The respondents who think AVs will have a significantly better effect on traffic injuries has no change in the opinion. Furthermore, a greater proportion of those who agree to share AV performance data with proper authorities had a negative opinion, while whose who are unsure or do not want release of such data had no change in the opinion. Middle aged respondents (25–34) had a greater proportion (27.4%) of negative opinions, while 35–44 age group had a greater proportion of positive opinion (28.6%).

### 2.2 Statistical analysis

A total of 796 responses were received from BikePGH members and public. In this study, only 726 complete responses were analyzed. The remaining incomplete responses were excluded to ensure data consistency and reliability in our analysis.

The impact of operational competence of the AV manufacturers on participants' safety perception was first studied. In this study, *perceived operational competence* is defined as the belief that AV manufacturers can effectively manage AVs during a trip, considering safety, performance and other important information [13,27]. Variables that measure the operational competence were identified, and Cronbach's alpha was computed to measure the internal consistency i.e., to check how closely related these variables are as a group [28]. A one-way analysis of variance (ANOVA) test was used to determine the difference between the average safety perception of non-motorists across the different levels of operational competence measures. Furthermore, an investigation was performed using Turkey Honest Significant Differences (HSD) test to examine where the difference lies [29,30].

Next, an analysis was performed to understand the factors responsible for the safety perception of non-motorists with different opinion positions after the crash occurrence. The Arizona crash had a varied effect on non-motorists’ opinion. While some studies have shown that crashes involving AVs could have a negative impact on public's receptivity [31], the data showed that some respondents' opinion shifted to a more positive outlook. This is understandable, as people may believe that crashes will encourage manufacturers to develop better safety technologies for AVs. Due to the limited number of observations for respondents who had a positive opinion after the crash, the study of the safety perception of non-motorists whose opinion was impacted by the crash focuses exclusively on those with a negative opinion.

The data was split into two sets i.e., non-motorists whose opinion was impacted after the crash and non-motorists whose opinions remained the same. Two separate five-ordered-response models were developed for the respondents whose opinions changed negatively and the respondents who had no change in the opinion due to the crash. The five ordered dependent variable levels range from 1 to 5, with 1 being very unsafe and 5 being very safe. The dependent variable was considered as an ordered variable, therefore, warranting the need for an ordinal logit model [32]. A proportional odds model, which is a type of ordinal logit model, was used in this study.

Following a crash, non-motorists’ opinion about AVs can sway from positive to negative or remain the same. The point of interest is in understanding how the safety perception among these individuals varies (i.e., non-motorists with negative opinions and non-motorists whose opinions remain the same). The safety perception of AV is defined by how safe respondents feel sharing the road with AV.

Let Y represent the response variable, in this case, ‘non-motorists safety perception of AV’ with J factors. Then *P(Y ≤ j)* is the probability of Y being less than or equal to a specific level j = 1, …, J (i.e., 1 to 5; with 1 = very unsafe and 5 = very safe). The odds of being less than or equal to a specific level is defined as Equation (1).

(1) $$\frac{P\left(Y\leq j\right)}{P\left(Y>j\right)}$$

Since the *P(Y > j)* is zero, and having zero in the denominator is impossible. The log odd is, therefore, expressed as Equation (2).

(2) $$\log\frac{P\left(Y\leq j\right)}{P\left(Y>j\right)}=logit\left(P\left(Y\leq j\right)\right)$$

The partial proportional odds can be parametrized as Equation (3).

(3) $$logit\left(P\left(Y\leq j\right)\right)=\alpha_{j_{0}}-\beta_{1}x_{1}\ldots -\beta_{p}x_{p}$$

where $\alpha_{j_{0}}$ is the model intercept, $\beta_{1}\ldots \;\beta_{p}$ are model coefficient parameters representing the slopes with p independent variables for j = 1, 2 …, (J - 1).

A Chi-Square test was applied to test for evidence of relationship among independent variables as the proportional odds model is not robust against multicollinearity among independent variables [32]. It was employed because all the independent variables are categorical [33]. However, only strong evidence of relationship among independent variables was treated as highly correlated.

Only one independent variable was included in the model if there is strong evidence of a relationship among two or more independent variables. Ordinal independent variables are modeled as orthogonal polynomial to retain the information in the rank [34]. Further analysis was done by conducting a backward stepwise multivariate ordinal logit model. The best model with a good predictive strength of McFadden's pseudo-R-square between 0.2 and 0.4 was selected, and the parallel slope assumption between the response level was checked [35,36]. Brant-Wald test was used to screen out variables that violate this assumption. A significant p-value (*<0.05*) in this test indicates that the coefficient does not satisfy the parallel slope assumption [36]. R statistical tool was used to carry out the analyses.

The shortcoming of the above method is the assumption of fixed coefficient for all individuals in the samples. This would call for the need to account for heterogeneity of individuals. The random parameter approach was, therefore, adopted to accommodate this heterogeneity. The utility function of each individual is taken as a random variable such that the utility of the nth individual is described as Equation (4).

(4) $$U_{inj}=V_{njt}+\varepsilon_{njt}$$

where n is the number of individuals, j is the number of levels of dependent variable Y, and x is the number of choices. $V_{njt}$ represents the utility defined by the attributes for alternative j in x choices and $\varepsilon_{njt}$ represents the error term over alternative choices. In this model, the maximum likelihood estimation is given as the conditional probability of the choice made by every individual, and it is expressed as Equation (5).

(5) $$S_{n}\left(\beta_{n}\right)=\prod_{x}L_{nj\left(n,x\right)x}\left(\beta_{n}\right)$$

where nj(n,x) is the alternative choices of individual n in x choices. The probability for this is given as a function *P*_*n*_*(θ)* that needs to be simulated over some random draws of β from a density function *f(βn |θ)*. The probability function is expressed as Equation (6).

(6) $$P_{n}\left(\theta \right)=\int S_{n}\left(\beta_{n}\right)\;f\left(\beta_{n}|\;\theta \right)d\beta_{n}$$

Halton draw was used since they provide a more efficient simulation for random parameter model estimation. Marginal effects of the random parameter ordered logit model were computed to measure the influence of a variable on a safety perception level while keeping other variables constant. An analysis of deviance test was carried out to compare the two model types (i.e., fixed parameter versus random parameter ordered logit model) for evidence of difference between both the models, for both the respondents with negative opinion and the respondents with no change in the opinion after hearing about the crash in Arizona.

## 3 Results & discussion

As stated previously, 726 complete responses were included in the analysis while the remaining 69 incomplete responses were excluded to ensure data consistency and reliability of the results. Of the 726 complete responses, 365 respondents are BikePGH members, 293 respondents are the general public (non-members), and 68 respondents were uncertain about their membership status with BikePGH. For the purpose of analysis, the responses from those uncertain about their membership status were combined with the responses from the general public.

**Fig. 1:**
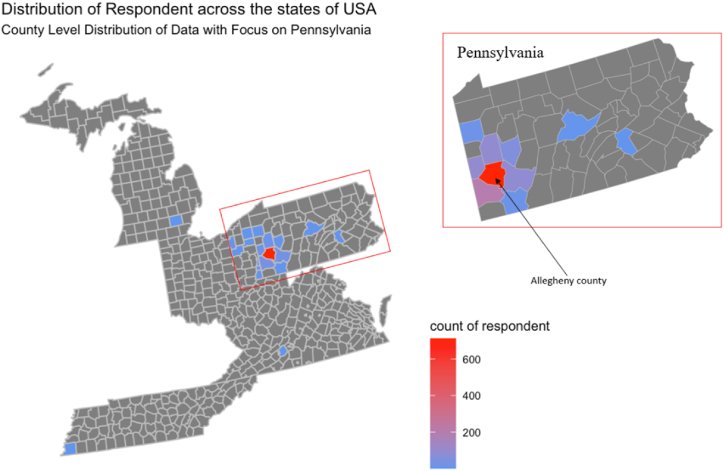
Spatial distribution of respondents.

The distribution of the survey responses across counties in Pennsylvania was verified using the R statistical software. They are mapped using the zip code and summarized at the county level. Fig. 1 illustrates the count of respondents in each county. Visual inspection of the map reveals a spatial clustering of respondents primarily around Allegheny County, indicating that a significant proportion of respondents resides near the Pittsburgh area and may have had interactions with AVs on the proving ground. There were also a small number of respondents from neighboring states, including Michigan, Ohio, Tennessee, Virginia, and West Virginia.

### 3.1 Non-motorist's perceived operational competence of AV manufacturers

The variables reflecting the operational performance of AV manufacturers were used to assess non-motorists’ perceived operational competence of AV manufacturers and its influence on safety perception. These variables include the belief that AV manufacturers should report safety concerns, share trip information, and share AV performance data. The results of Cronbach's alpha coefficient suggest that these three variables have an acceptable internal consistency (α = 0.708). Table 3 summarizes the findings, which indicate that the respondents with the higher perceived operational competence of AV manufacturers are more likely not to support the sharing of AV data with proper authorities.

**Table 3:** Non-motorist's perceived operational competence of the AV manufacturers.

Question	Levels	Safety Perception of AV					Mean	F- statistic
		1 = very unsafe	2	3	4	5 = very safe		
Share trip	No	2.0 (4.1%)	5 .0 (8.5%)	8 .0 (4.8%)	28.0 (12.1%)	48.0 (21.8%)	4.2	15.7***
	Not sure	7.0 (14.3%)	3.0 (5.1%)	17.0 (10.2%)	26.0 (11.2%)	38 .0 (17.3%)	3.9	
	Yes	40.0 (81.6%)	51.0 (86.4%)	141.0 (84.9%)	178.0 (76.7%)	134.0 (60.9%)	3.6	
Report safety concerns to the authorities	No	2.0 (4.1%)	0.0 (0.0%)	5.0 (3.0%)	46.0 (6.9%)	39.0 (17.7%)	4.4	19.3***
	Not sure	1.0 (2.0%)	1.0 (1.7%)	8.0 (4.8%)	19.0 (8.2%)	20.0 (9.1%)	4.1	
	Yes	46.0 (93.9%)	58.0 (98.3%)	153.0 (92.2%)	197.0 (84.9%)	161.0 (73.2%)	3.6	
Share AV performance data	No	2.0 (4.1%)	0.0 (0.0%)	8.0 (4.8%)	12.0 (5.2%)	36.0 (16.4%)	4.4	15.5***
	Not sure	0.0 (0.0%)	1.0 (1.7%)	9.0 (5.4%)	21.0 (9.1%)	18.0 (8.2%)	4.1	
	Yes	47.0 (95.9%)	58.0 (98.3%)	149.0 (89.8%)	199.0 (85.8%)	166.0 (75.5%)	3.6	

N.B: *indicate significant F-statistic with p-value <0.05; **indicate significant F-statistic with p-value <0.01; *** indicate significant F-statistic with p-value <0.001.

A higher proportion of respondents who were unsure or opposed to AV manufacturers sharing trip information with the proper authorities feel very safe about sharing the road with the AVs. In contrast, a higher proportion of respondents who supported the sharing of trip information with AV manufacturers reported feeling unsafe about sharing the road with the AVs. This finding suggests a potential disconnect between respondents' perceptions of safety and their stance on trip information sharing. It is important to note that further research is required to understand the underlying reasons behind this association. Possible explanations could include concerns about privacy, trust in the AV technology, or differing beliefs regarding the effectiveness of trip information sharing in enhancing the safety.

The pattern is similar for non-motorist's desire for the AV manufacturers to report safety concerns to the proper authorities. A higher proportion of respondents who are unsure or opposed to AV manufacturers reporting safety concerns to the proper authorities feel safe sharing the road with the AVs. Conversely, a higher proportion of respondents who want AV manufacturers to report safety concerns to proper authorities feel unsafe about sharing the road with the AVs. This result implies that the respondents feel safer sharing the road with the AVs knowing that there is better management of AV operations which can ultimately improve safety.

A substantial proportion of the respondents, including those uncertain about the AV performance data sharing with proper authorities, expressed a high level of confidence in sharing the road with the AVs. However, a higher proportion of the respondents who want trip information to be shared with the AV manufacturers feel unsafe sharing the road with the AVs. In general, higher number of respondents who support the openness of the AV manufacturers about data have lower safety perceptions of the AVs. This result implies that respondents who have less trust in the AV manufacturers have a lower safety perception of the AVs while the respondents who are confident in the AV manufacturers have a higher safety perception of the AVs.

The one-way ANOVA result revealed a difference in the safety perception and showed a difference between the safety perception of the respondents who wanted trip, safety, and performance data to be shared with the proper authorities. The respondents who do not support the AV manufacturers sharing data with the proper authorities feel safer sharing the road with the AV compared to the respondents who want data information to be made available to the proper authorities.

Turkey HSD was conducted to investigate where these significant differences occurred. The result shows that the only difference between the respondents who do not want data to be shared and those who are not sure about data transparency is insignificant across all the three variables that measure the operational competence of the AV manufacturers.

Overall, the result shows that respondents who are more confident in the AV manufacturers and their operational competence tend to have a higher safety perception of the AVs, while those who are uncertain or less trusting of the AV manufacturers tend to be less willing to share the road with the AVs. This suggests that the trust the public have in AV manufacturers is directly related to the public's willingness to share the road with the AVs.

Two proportional odds models were developed using R statistical software to investigate the factors associated with the respondents' safety perception of non-motorists whose opinions were influenced hearing about the crash in Arizona. Independent variables with high correlation were eliminated via correlation matrix constructed from a Chi-Square test. Brant-Wald test was conducted to confirm the need for a proportional odds model. The result shows that the future impact of the AVs on traffic injuries and the use of Pittsburgh streets as a proving ground for the AVs are associated with the respondent's safety perception. McFadden's pseudo-R-square of 0.344 shows that the model has good predictive strength.

### 3.2 Effect of explanatory variables on the safety perception of respondents exhibiting a negative change in opinion

From Table 4, there is a higher likelihood of having a safe perception of AVs as the future impact of the AVs on traffic injuries increases from significantly worse to significantly better. However, as the impact of the AVs on traffic injuries decreases from significantly worse to significantly better, there is a decline in the probability of classifying it as very unsafe or unsafe. This finding suggests that the respondents' perception of AV safety is influenced by their expectations regarding the impact of AVs on reducing traffic injuries. This result is consistent with findings reported in the existing literature [12,22]. Several studies have highlighted the importance of perceived safety in shaping public acceptance and trust towards the AV technology [37,38]. When individuals perceive AVs to have the potential to reduce traffic injuries and improve overall safety, they tend to view them more favorably.

**Table 4:** Results of ordered logit model examining non-motorists' safety perception among respondents who experienced negative or no change in opinion following the crash.

Variable	Level	Negative Change					No Change					Ratio of Fixed β
		Fixed Parameter			Random Parameter		Fixed Parameter			Random Parameter		
		β	Wald Chi-sq.	OR	β	Wald Chi-sq.	β	Wald Chi-sq.	OR	Random β	Wald Chi-sq.	
Thresholds	1 = very unsafe | 2	1.668	–	–	1.724	–	15.600***	–	–	–	–	0.107
	2 | 3	4.029***	–	–	4.122**	–	17.053***	–	–	–	–	0.236
	3 | 4	7.051***	–	–	7.055**	–	19.772***	–	–	–	–	0.357
	4 | 5 = very safe	10.027***	–	–	9.862**	–	22.006	–	–	–	–	0.456
Report safety concerns to the authorities	No	Ref	–		–		Ref	–	–	–		
	Not sure	−1.420	0.000	0.241	−1.451	0.000	−0.448	5.27	0.639	−0.171	5.022	3.170
	Yes	−0.981	0.75	0.375	−1.136	0.000	−0.714*	4.58	0.489	−0.275	4.369	1.374
	s.d- Yes									1.095*	0.234	
Impact of AV	Significantly worse	Ref	–	–	–		Ref	–	–	–	–	–
	Slightly worse	1.308*	4.610	3.699	1.452*	4.328	15.811***	0.000	7.361e06	15.602***	0.000	0.083
	No effect	2.710***	1.520	15.028	2.820**	1.413	17.880***	0.000	5.824e07	16.697***	0.000	0.152
	Slightly better	3.281***	0.000	26.610	3.286**	0.000	18.639***	0.000	1.243e08	18.094***	0.000	0.176
	Significantly better	4.883***	0.190	131.968	5.221**	0.049	19.673***	0.000	3.499e08	19.508***	0.000	0.245
Pittsburgh public street as a proving ground	Disapprove	Ref	–	–	–	–	Ref	–	–	–	–	
	Somewhat disapprove	2.320***	8.760	10.172	2.851***	8.258	1.893	0.000	6.641	1.661	0.000	1.226
	Neutral	3.134***	7.530	22.973	3.564***	7.066	2.684*	0.390	14.651	2.579*	0.111	1.168
	Somewhat approve	3.697***	19.240	40.341	3.665***	18.962	2.671*	0.000	14.460	2.547 **	0.000	1.384
	Approve	4.763***	15.210	117.125	4.881***	15.089	3.612***	2.130	37.060	3.503***	3.601	1.319
Share trip data with authorities	No	Ref	–	–	–	–	Ref	–	–	–	–	–
	Not sure	−0.384	2.700	0.681	−0.582	2.308	0.050	4.990	1.052	0.053	4.662	−7.680
	Yes	0.076	5.820	1.079	0.126	5.227	−0.517	7.340	0.596	−0.557	6.972	−0.147
Manual mode of AV in school zone	No	Ref	–	–	–	–	Ref	–	–	–		
	Not sure	0.554	6.110	1.740	0.982*	7.486	−0.311	1.530	0.733	−0.275	1.072	−1.781
	s.d- Not sure				1.344**	0.562						
	Yes	0.454	6.190	1.575	0.533	10.639	−0.546*	0.740	0.579	−0.541*	0.000	−0.832
Familiarity with AV Tech	Not familiar at all	Ref	–	–	–	–	Ref	–	–	–	–	–
	Somewhat familiar	0.440	2.130	1.553	0.529*	1.335	0.116	3.830	1.123	0.103	3.821	3.793
	s.d- Somewhat familiar				0.365*	5.324						
	Mostly familiar	0.554	4.890	1.740	0.647	2.937	0.926*	1.050	2.524	0.857*	0.946	0.598
	Extremely familiar	0.823	1.460	2.278	1.626*	0.000	0.623	0.400	1.864	0.594	0.000	1.321
	s.d- Extremely familiar				2.439*	3.777						
Number of Observations		263			263		442			442		
Log-Likelihood		−254.032			−253.940		−422.889			−418.332		
McFadden's R^2^		0.353			0.357		0.236			0.256		
Akaike Information Criterion		550.064			548.331		887.777			880.672		

N.B: *indicate p-value <0.05; **indicate p-value <0.01; *** indicate p-value <0.001.

The more the respondents are willing to approve Pittsburg streets as a proving ground for AVs, the higher the likelihood of identifying as having a 'very safe' or 'safe' perception of the AVs. Conversely, the higher the likelihood of identifying as 'very unsafe' or 'unsafe' perception of the AVs the more they disapprove. This finding aligns with prior studies indicating that negative attitudes towards the testing environment are associated with heightened concerns regarding the safety of AVs [20,22]. The findings suggest that efforts to promote public acceptance of the AVs should not only focus on technological advancements but also consider the public's willingness to support testing initiatives and their perceived safety implications. Effective communication, engagement, and transparency in AV testing processes can help address public concerns and shape more positive perceptions of the AV safety.

In general, the interaction of the future impact of AVs on traffic injuries and the respondent's willingness to approve Pittsburg streets as a proving ground increases the likelihood of identifying as having a very safe perception of AVs for the respondents with a negative opinion hearing about the crash in Arizona.

### 3.3 Effect of explanatory variables on the safety perception of the respondents exhibiting no change in opinion

A separate proportional odds model was developed to explore the factors associated with safety perception of non-motorists whose opinion was unaffected hearing about the Arizona crash. Six variables i.e., the impact of AVs on traffic injuries, use of Pittsburgh streets as a proving ground, familiarity with the AVs, reporting safety concerns to proper authorities, sharing trip information with the authorities, and manual operation in school environments were found to influence the safety perception. Brant-Wald test validated the need for the model [36], with strong predictive strength as indicated by a McFadden's pseudo-R-square of 0.236 (Table 4).

Specifically, the respondents who expressed uncertainty about whether AV manufacturers should report safety concerns to the proper authorities, as opposed to those who do not support such reporting, are associated with a lower likelihood of having a higher safety perception of the AVs. This suggests that their indecision or lack of clarity on the reporting issue may contribute to a more cautious or less positive view of the AV safety. Similarly, respondents who express a desire for AV manufacturers to report safety concerns about the AVs to the proper authorities, compared to those who do not support reporting, also have a lower likelihood of having a higher safety perception of the AVs. This finding aligns with the study of Hulse et al. [16] which suggests that even among non-motorists who exhibit no change in opinion after an AV crash, those who advocate for reporting safety concerns may still harbor concerns or doubts about the overall safety of the AV technology. These findings further elucidate on the influence of individuals' attitudes toward reporting safety concerns on their perception of the AV safety. It suggests that the perception of a proactive and transparent reporting system by AV manufacturers may play a role in shaping non-motorists' safety perceptions [39]. Therefore, considering and addressing concerns related to safety reporting protocols could be important in fostering positive safety perceptions and gaining trust among non-motorists in the context of AV technology.

When examining the effect of future impact of traffic injuries on the respondents safety perception of AVs among non-motorists whose opinion remains unchanged hearing about the crash, there is a higher likelihood of having a safer perception of AVs as they think that the future impact of AVs on traffic injuries changes from significantly worse to significantly better. This implies that non-motorists feel safe sharing the road with the AVs knowing that it will reduce traffic injuries. Rahman et al. [22] in their study argued that non-motorists' anticipation of fewer traffic injuries with the widespread use of AVs could bolster their confidence in sharing the road with these vehicles.

The more the respondents are willing to approve Pittsburg streets as a proving ground for AVs, the higher the likelihood of identifying as having a 'very safe' or 'safe' perception of AVs. This result is similar to the findings of Penmetsa et al. [12]. It implies that non-motorists feel safer sharing the road with AVs as they become more willing to approve Pittsburgh streets as a testing ground for the AVs. Perhaps, the experience of sharing the road with the AVs improves the safety perception of non-motorists.

The respondents who are unsure about their desire for the AV manufacturers to share trip information as opposed to respondents who do not want the AV manufacturers to share trip data with the proper authorities are associated with a slightly higher likelihood of having a higher safety perception of the AVs. However, the respondents who want trip information to be shared as opposed to those who do not want the AV manufacturers to share trip information are associated with a lower likelihood of having a safer perception of the AVs. While some non-motorists feel better knowing that authorities (say, departments of transportation) have access to their trip information to provide better travel information, others are more concerned about their safety knowing that the AV manufacturers can sell out their trip information.

The respondents who are unsure about AVs operating in manual operation while in school zones compared to those who do not want it to operate in manual mode while in school zones are associated with a lower likelihood of having a higher safety perception of the AVs. Likewise, the respondents who want AVs to operate in manual modes while in a school zone as opposed to respondents who do not want AVs to operate in manual mode while in a school zone are associated with a significantly lower likelihood (*p < 0.05*) of having a higher safety perception of the AVs.

The likelihood of having a safe perception of AVs increases if respondents familiarity with the AV technology increases. This implies that the more familiar non-motorists are with the AV technology, the more likely they would want to share the road with it.

### 3.4 Difference across respondents exhibiting no change and negative change in opinion

The focus of this study is also in comparing how the effect of explanatory variables differ across groups: i.e., is the effect of familiarity with AVs on non-motorist's safety perception greater for respondents with no change than it is for respondents with negative change? From Table 4, the respondents with a negative change and who are not sure about the AV manufacturers reporting safety concerns to the proper authorities are three times less likely to feel safe sharing the road with the AVs compared to the respondents with no change in the opinion after hearing about the crash. However, the respondents with a negative change and who are sure about the AV manufacturers reporting safety concerns to the proper authorities are 1.37 times less likely to feel safe sharing the road with the AVs compared to respondents with no change in the opinion after hearing about the crash. The difference suggests that the respondents with no change in the opinion after hearing about the crash have a higher safety perception about sharing the road with the AVs as they think safety concern should be reported to the proper authorities. This result seems reasonable enough in the sense that the respondents with a negative change in the opinion about AVs after the crash have a higher safety concern about the AVs. This group of people do not trust the AV manufacturers to manage safety related data without the involvement of proper authorities.

Contrarily, the respondents with a negative change and who are extremely familiar with the AV technology are 1.32 times more likely to feel safer sharing the road with the AVs compared to the respondents with no change in the opinion after the crash. The difference suggests that non-motorists who are extremely familiar with the AVs have higher safety perception for the negative change in the opinion compared to the no change in the opinion, which may sound counterintuitive. This might be because of the difference in residual variances. Perhaps, the respondents with a negative change in the opinion of AVs after hearing about the crash have a more heterogeneous behavior, or maybe some unmeasured variables are affecting the safety perception of the respondents with no change than for the respondents with negative change in the opinion.

The coefficient of respondent's familiarity with the AV technology is zero (not significant). This implies that the familiarity with the AV technology is not important for non-motorist's safety perception. The random parameter approach was used to address this issue of difference in residual variance. Table 4 also summarizes the random parameter estimates and the corresponding Wald Chi-Square statistics.

### 3.5 Random parameter model to account for individual differences among respondents

The results from the random parameter ordered logit model provide insights into the heterogeneity of respondents' behavior and the variable effects on the safety perception of the AVs among non-motorists with a negative change in opinion hearing about the crash. The model was estimated through simulated maximum likelihood of 200 Halton draws. A normal distribution was assumed for random parameter analysis. The best subset selection method was used for variable selection with a criterion of p-value <0.05. Just like the ordered logit model with fixed parameters, the variables found to be statistically significant in both models for negative change and no change in the opinion were different. Table 5 shows the marginal effect of the random parameter ordered logit model.

**Table 5:** Marginal effect from the random parameter ordered logit model.

Variable	Level	Negative Change					No Change				
		1	2	3	4	5	1	2	3	4	5
Report safety concerns to the authorities	No	Ref	–	–	–	–	Ref	–	–	–	–
	Not sure	0.026	0.174	0.019	−0.203	−0.017	0.002	0.005	0.065	0.022	−0.094
	Yes	0.018	0.120	0.013	−0.140	−0.011	0.003	0.008	0.103	0.035	−0.150
Impact of AV	Significantly worse	Ref	–	–	–	–	Ref	–	–	–	–
	Slightly worse	−0.024*	−0.160*	−0.018	0.187*	0.015*	−0.056*	−0.178*	−2.112	−0.721*	3.068*
	No effect	−0.051**	−0.332***	−0.037	0.388***	0.032**	−0.064**	−0.203**	−2.411	−0.823	3.501***
	Slightly better	−0.061**	−0.403***	−0.045	0.470***	0.039**	−0.067	−0.213	−2.520	−0.861	3.660
	Significantly better	−0.091**	−0.600***	−0.066	0.699***	0.058**	−0.071	−0.225	−2.669	−0.911	3.877
Pittsburgh public street as a proving ground	Disapprove	Ref	–	–	–	–	Ref	–	–	–	–
	Somewhat disapprove	−0.043**	−0.285***	−0.031	0.332***	0.027**	−0.007*	−0.023**	−0.2733	−0.093*	0.397**
	Neutral	−0.058**	−0.385***	−0.043	0.449***	0.037**	−0.010*	−0.033	−0.387	−0.132	0.563
	Somewhat approve	−0.068**	−0.454***	−0.050	0.529***	0.044**	−0.010	−0.033**	−0.386	−0.132	0.560
	Approve	−0.089**	−0.585***	−0.064	0.682***	0.056**	−0.014*	−0.044***	−0.521	−0.178	0.757
Share trip data with authorities	No	Ref	–	–	–	–	Ref	–	–	–	–
	Not sure	0.007	0.047	0.005	−0.055	−0.005	−0.000	−0.001	−0.007	−0.002	0.011*
	Yes	−0.001	−0.009	−0.001	0.011	0.001	0.002	0.006	0.075	0.025*	−0.108
Manual mode of AV in school zone	No	Ref	–	–	–	–	Ref	–	–	–	–
	Not sure	−0.010	−0.068	−0.008	0.079	0.007	0.001	0.004	0.045	0.015	−0.065
	Yes	−0.008	−0.056	−0.006	0.065	0.005	0.002	0.007	0.079	0.027**	−0.115*
Familiarity with AV Tech	Not familiar at all	Ref	–	–	–	–	Ref	–	–	–	–
	Somewhat familiar	−0.008	−0.054	−0.006	0.063	0.005	−0.000	−0.001	−0017	−0.006	0.024
	Mostly familiar	−0.010	−0.068	−0.008	0.079	0.006	−0.004	−0.011	−0.134*	−0.046	0.194
	Extremely familiar	−0.015	−0.101	−0.011	0.118	0.010	−0.002	−0.007	−0.090	−0.031**	0.131

N.B: *indicate significant p-value <0.05; **indicate significant p-value <0.01; *** indicate significant p-value <0.001.

The familiarity with AV technology is found to have a variable effect on safety perception. Specifically, respondents who reported being somewhat familiar and those who reported being extremely familiar with the AV technology exhibited significant differences in their safety perceptions. This suggests that individuals with varying degrees of familiarity may interpret and evaluate the AV safety differently, leading to different perceptions of safety. Othman [25] adjusted for individual difference by accounting for demographic characteristics. It was argued that familiarity does not necessarily translate to optimism among the public. This study supports the argument by further showing how the same level of familiarity among non-motorists can lead to different safety perception.

The indicator for respondents who expressed uncertainty about whether AVs should operate in manual operation while active in school zones is also found to be significant. This indicates that the level of certainty or opinion regarding manual operation in school zones plays a role in shaping safety perceptions. The respondents who are unsure about this aspect may have greater variability in their safety perceptions compared to those who hold a clear stance. The presence of random parameters indicates the existence of heterogeneity in the way individuals form their perceptions.

The model estimation results for respondents exhibiting no change in opinion after the crash indicate that only one variable has a random parameter and affects the safety perception of non-motorists. This suggests that there is less heterogeneity in the factors influencing safety perception among this group of respondents compared to those with a negative change in opinion.

Separate models based on respondents with negative change and no change in the opinion after hearing about the crash in Arizona shed more light on identifying contributing factors responsible for their respective safety perception of the AVs. However, a limitation of difference in residual variance among individual was identified and solved by introducing a random parameter. An analysis of deviance test shows that the random parameter ordered logit model is significantly different from the fixed parameter ordered logit model.

## 4 Conclusions & recommendations

The study has both theoretical and practical implications. It adds to the existing knowledge of AV perception research by studying how the non-motorist's perceived operational competence of the AV manufacturers (trip data, safety concerns and performance data) affect their safety perception of the AVs. While other studies have researched the role of trust in the adoption of AVs, they have not studied if the public would trust them enough to manage the data of AVs. In this study, the operational competence of the AV manufacturers was measured using three variables i.e., respondent's desire for the AV manufacturers to share AV trip, performance, and safety concerns. The result shows that the respondents who have high perceived operational competence of the AV manufacturers have higher safety perception of the AVs while the respondents who have low perceived operational competence of the AV manufacturers have lower safety perception of the AVs. This suggests that non-motorists need to have more trust in the AV manufacturers before they are willing to share the road with them. To increase non-motorists’ trust and acceptance of the AVs, policymakers should consider implementing initiatives that promote the perceived operational competence of the AV manufacturers. This could include introducing legislations that requires the AV manufacturers to report safety concerns, share trip information, and share AV performance data. Additionally, policymakers should provide incentives to the AV manufacturers to ensure that they are compliant with such regulations. Through such initiatives, policymakers would be able to build non-motorists’ trust in the AV manufacturers and thus increasing their acceptance of the AVs.

The study also investigates how non-motorists whose opinions swayed differ from those whose opinions remained the same after a crash. For non-motorists whose opinions changed negatively after hearing about a crash, their perceived willingness to share the road with AVs is influenced by their assessment of the future impact of AVs and their desire for streets to be used as a proving ground for AVs. However, non-motorists whose opinion remains unchanged after hearing about a crash have additional factors influencing their perceived willingness to share the road with the AVs. The results suggest that negative information about the crash may have an effect on the perceptions of AVs, but this effect can be mitigated by providing positive information about the AVs. It is recommended that further research be conducted to explore the effects of positive versus negative information on people's perceptions of the AVs. Additionally, it is recommended that AV manufacturers and policymakers consider the implications of the study results in order to ensure that the public is adequately informed about the AV technology and its implications. Finally, it is recommended that AV manufacturers and policymakers strive to create an environment that is conducive to public dialogue on the issue of AVs. This could involve providing more public forums, such as town hall meetings, to allow members of the public to voice their questions and concerns about the AVs.

A few non-motorist perception studies were carried out in recent times [12,21,40,41]. These studies used data that were collected pre-COVID, just like in this study. Therefore, there is a need to conduct a post-COVID survey to analyze how the pandemic had influenced non-motorists' perception of AV over time.

## Author contribution statement

Abimbola Ogungbire: Conceived and designed the experiments; Performed the experiments; Analyzed and interpreted the data; Wrote the paper.

Srinivas Subrahmanyam Pulugurtha: Conceived and designed the experiments; Contributed reagents, materials, analysis tools or data; Analyzed and interpreted the data; Wrote the paper.

## Data availability statement

Data associated with this study has been deposited at (https://data.wprdc.org/dataset/autonomous-vehicle-survey-of-bicyclists-and-pedestrians.

## Declaration of competing interest

The authors declare that they have no known competing financial interests or personal relationships that could have appeared to influence the work reported in this paper.

## References

[1] Baron O., Berman O., Nourinejad M. (2018).

[2] Mathew S., Duvvuri S., Pulugurtha S.S. (2020). International Conference on Transportation and Development 2020.

[3] Othman K. (2021). Public acceptance and perception of autonomous vehicles: a comprehensive review. AI and Ethics.

[4] Fagnant D.J., Kockelman K.M. (2018). Dynamic ride-sharing and fleet sizing for a system of shared autonomous vehicles in Austin, Texas. Transportation.

[5] Forrest A., Konca M. (2007).

[6] Reimer B. (2014). Driver assistance systems and the transition to automated vehicles: a path to increase older adult safety and mobility?. Public Policy & Aging Report.

[7] National Highway Traffic Safety Administration (NHTSA) (2017). https://www.nhtsa.gov/sites/nhtsa.gov/files/documents/13069a-ads2.0_090617_v9a_tag.pdf?xid=PS_smithsonian.

[8] Bertoncello M., Wee D. (2015). https://www.mckinsey.com.br/%7E/media/McKinsey/Industries/Automotive.

[9] Liu P., Yang R., Xu Z. (2019). Public acceptance of fully automated driving: effects of social trust and risk/benefit perceptions. Risk Anal..

[10] Salonen A.O. (2018). Passenger's subjective traffic safety, in-vehicle security and emergency management in the driverless shuttle bus in Finland. Transport Pol..

[11] Cartenì A. (2020). The acceptability value of autonomous vehicles: a quantitative analysis of the willingness to pay for shared autonomous vehicles (SAVs) mobility services. Transp. Res. Interdiscip. Perspect..

[12] Penmetsa P., Adanu E.K., Wood D., Wang T., Jones S.L. (2019). Perceptions and expectations of autonomous vehicles–A snapshot of vulnerable road user opinion. Technol. Forecast. Soc. Change.

[13] Bansal P., Kockelman K.M. (2017). Forecasting Americans' long-term adoption of connected and autonomous vehicle technologies. Transport. Res. Pol. Pract..

[14] BikePGH (2019). http://www.bikepgh.org/resources/save/survey/.

[15] Choi J.K., Ji Y.G. (2015). Investigating the importance of trust on adopting an autonomous vehicle. Int. J. Hum. Comput. Interact..

[16] Hulse L.M., Xie H., Galea E.R. (2018). Perceptions of autonomous vehicles: relationships with road users, risk, gender and age. Saf. Sci..

[17] Hurst K.F., Sintov N.D. (2022). Trusting autonomous vehicles as moral agents improves related policy support. Front. Psychol..

[18] Du M., Zhang T., Liu J., Xu Z., Liu P. (2022). Rumors in the air? Exploring public misconceptions about automated vehicles. Transport. Res. Pol. Pract..

[19] Liu P., Du M., Xu Z., Chu Y. (2022). People with more misconceptions about automated vehicles might be more positive toward them. Transport. Res. F Traffic Psychol. Behav..

[20] Das S., Sheykhfard A., Liu J., Khan M.N. (2023). Understanding non-motorists' views on automated vehicle safety through Bayesian network analysis and latent Dirichlet allocation. International Journal of Transportation Science and Technology.

[21] Xing Y., Zhou H., Han X., Zhang M., Lu J. (2022). What influences vulnerable road users' perceptions of autonomous vehicles? A comparative analysis of the 2017 and 2019 Pittsburgh surveys. Technol. Forecast. Soc. Change.

[22] Rahman M.T., Dey K., Pyrialakou V.D., Das S. (2023). Factors influencing safety perceptions of sharing roadways with autonomous vehicles among vulnerable roadway users. J. Saf. Res..

[23] Cugurullo F., Acheampong R.A., Gueriau M., Dusparic I. (2021). The transition to autonomous cars, the redesign of cities and the future of urban sustainability. Urban Geogr..

[24] Sankeerthana G., Raghuram Kadali B. (2022). A strategic review approach on adoption of autonomous vehicles and its risk perception by road users. Innovative Infrastructure Solutions.

[25] Othman K. (2023). Public attitude towards autonomous vehicles before and after crashes: a detailed analysis based on the demographic characteristics. Cogent Engineering.

[26] Othman K. (2023). A microscopic analysis of the relationship between prior knowledge about self-driving cars and the public acceptance: a Survey in the US. Transport and Telecommunication Journal.

[27] Buell R.W., Choi M. (2019).

[28] Tavakol M., Dennick R. (2011). Making sense of Cronbach's alpha. Int. J. Med. Educ..

[29] Sportillo D., Paljic A., Ojeda L. (2018). Get ready for automated driving using virtual reality. Accid. Anal. Prev..

[30] St L., Wold S. (1989). Analysis of variance (ANOVA). Chemometr. Intell. Lab. Syst..

[31] Wang S., Li Z. (2019). Exploring the mechanism of crashes with automated vehicles using statistical modeling approaches. PLoS One.

[32] Williams R. (2016). Understanding and interpreting generalized ordered logit models. J. Math. Sociol..

[33] Sucky R.N. (2022). Chi-square test for correlation test in details: manual and Python implementation. Towards Data Sci..

[34] Lombardo R., Beh E.J., D'Ambra A. (2011). Studying the dependence between ordinal-nominal categorical variables via orthogonal polynomials. J. Appl. Stat..

[35] Houseal L.A., Gaweesh S.M., Dadvar S., Ahmed M.M. (2022). Causes and effects of autonomous vehicle field test crashes and disengagements using exploratory factor analysis, binary logistic regression, and decision trees. Transport. Res. Rec..

[36] Grilli L., Rampichini C. (2014).

[37] Golbabaei F., Yigitcanlar T., Paz A., Bunker J. (2020). Individual predictors of autonomous vehicle public acceptance and intention to use: a systematic review of the literature. Journal of Open Innovation: Technology, Market, and Complexity.

[38] Zhang T., Tao D., Qu X., Zhang X., Lin R., Zhang W. (2019). The roles of initial trust and perceived risk in public's acceptance of automated vehicles. Transport. Res. C Emerg. Technol..

[39] Das S., Tsapakis I., Wei Z., Elgart Z., Kutela B., Vierkant V., Li E. (2022). http://hdl.handle.net/10919/112319.

[40] Das S., Dutta A., Fitzpatrick K. (2020). Technological perception on autonomous vehicles: perspectives of the non-motorists. Technol. Anal. Strat. Manag..

[41] Pyrialakou V.D., Gkartzonikas C., Gatlin J.D., Gkritza K. (2020). Perceptions of safety on a shared road: driving, cycling, or walking near an autonomous vehicle. J. Saf. Res..

